# Brazilian version of the Jefferson Scale of Empathy: psychometric properties and factor analysis

**DOI:** 10.1186/1472-6920-12-73

**Published:** 2012-08-09

**Authors:** Helena BMS Paro, Renata M Daud-Gallotti, Iolanda C Tibério, Rogério MC Pinto, Mílton A Martins

**Affiliations:** 1 Center for Development in Medical Education, University of Sao Paulo, Sao Paulo, Brazil; 2 Health Sciences, Federal University of Uberlandia, Uberlandia, Brazil

**Keywords:** Empathy, Students, Medical, Psychometrics

## Abstract

**Background:**

Empathy is a central characteristic of medical professionalism and has recently gained attention in medical education research. The Jefferson Scale of Empathy is the most commonly used measure of empathy worldwide, and to date it has been translated in 39 languages. This study aimed to adapt the Jefferson Scale of Empathy to the Brazilian culture and to test its reliability and validity among Brazilian medical students.

**Methods:**

The Portuguese version of the Jefferson Scale of Empathy was adapted to Brazil using back-translation techniques. This version was pretested among 39 fifth-year medical students in September 2010. During the final fifth- and sixth-year Objective Structured Clinical Examination (October 2011), 319 students were invited to respond to the scale anonymously. Cronbach’s alpha, exploratory factor analysis, item-total correlation, and gender comparisons were performed to check the reliability and validity of the scale.

**Results:**

The student response rate was 93.7% (299 students). Cronbach’s coefficient for the scale was 0.84. A principal component analysis confirmed the construct validity of the scale for three main factors: Compassionate Care (first factor), Ability to Stand in the Patient’s Shoes (second factor), and Perspective Taking (third factor). Gender comparisons did not reveal differences in the scores between female and male students.

**Conclusions:**

The adapted Brazilian version of the Jefferson Scale of Empathy proved to be a valid, reliable instrument for use in national and cross-cultural studies in medical education.

## Background

In a significant paradigm shift with respect to health, the World Health Organization proposed in 1946 [1] that empathy be considered a crucial skill to be developed in the context of medical education. In a holistic view of health, empathy is regarded as one of the central characteristics of medical professionalism and is frequently associated with the quality of care in clinical practice [2,3] as well as with improved health outcomes. Not surprisingly, medical schools and international educational councils [4] make constant efforts to foster medical students’ empathic skills.

In line with this trend, increasing numbers of studies have focused on empathy in medical education [5]. Such studies depend on the development of valid, reliable instruments for measuring empathy. The Jefferson Scale of Empathy (JSE) was designed to assess empathy in the context of patient care, and worldwide it is the most widely employed measure of empathy in medical education. It has been translated into 39 languages [6,7], and its psychometric properties have been confirmed in several countries [8-12].

A Portuguese version of the JSE (JSE-Pt) has been used in Portugal [13]. However, the JSE-Pt has not been tested to date in Brazil. Brazil is in fact the country with the second-largest number of medical schools in the world, with 185 institutions and approximately 15,000 final-year medical students annually. With more than 190 million inhabitants, Brazil is also the largest of the nine Portuguese-speaking countries in the world. Because there are important cultural differences between Portugal and Brazil, it is essential that the JSE-Pt be properly adapted for use in Brazil so as to produce an instrument that is as valid and reliable as the original. Such equivalence to the original version of the JSE can be assured through rigorous techniques of back-translation and the adaptation of questionnaires [14-17]. These procedures are especially important for meta-analytic studies that involve international educational commissions and are designed to implement and evaluate reform strategies in an interdependent world [18].

The aim of this study was to adapt the JSE-Pt to the Brazilian culture; we also aimed to test its reliability and validity so that we could produce a version that closely matched the original scale.

## Methods

### Setting

This study was conducted at the São Paulo University School of Medicine, which admits 180 students annually to a six-year program in undergraduate medical education, which ranges from primary to tertiary care. Clerkship experience occurs during the final two years of undergraduate education, and it involves supervised, hands-on training in two university hospitals of increasing complexity: a 258-bed secondary hospital and a 1,200-bed tertiary hospital. Students are divided into small groups and rotate among five main areas: pediatrics, internal medicine, obstetrics and gynecology, surgery, and preventive medicine. The internal medicine clerkship corresponds to a 12-week program for fifth-year students, during which small groups of students participate in supervised clinical activities in two internal medicine wards and ambulatory clinics. This program includes simulations of medical scenarios, which focus on effective interpersonal communication, invasive procedures, and resuscitation. Student performance is evaluated using the following three complementary tools: a supervisor’s overall rating, written exams, and an objective structured clinical examination (OSCE) with standardized patients.

### Participants

During the pretest phase, all fifth-year students taking the internal medicine clerkship OSCE in September 2010 at our institution were eligible for the study (*n* = 45).

Fifth- and sixth-year students taking the final OSCE in October 2011 were eligible for the validation phase of the study (*n* = 319).

After approval by the Ethics Committee of our institution, signed consent was obtained from all students. No reward was provided for participation.

### Instrument: JSE–student version (JSE-S)

The JSE is a self-administrated 20-item instrument designed to measure empathy in the patient-care context. Its original version comprises three domains: Perspective Taking (10 items), Compassionate Care (eight items), and Standing in the Patient’s Shoes (two items). The items are answered on a seven-point Likert scale from 1 (strongly disagree) to 7 (strongly agree) for positive items and 1 (strongly agree) to 7 (strongly disagree) for the 10 negative items; scores thus range from 20 to 140, with higher scores signifying greater empathy [3].

### Procedures

#### Phase 1: Cultural adaptation procedures

Because there is a validated Portuguese version of the JSE for use in Portugal (JSE-Pt) [13], we decided to follow international recommendations for cultural adaptation of questionnaires [17] using this existing version. The JSE-Pt was obtained from the authors and reviewed by three Brazilian translators to identify terms or usages that could be unacceptable or misunderstood in Brazil. A fourth reviewer analyzed the translators’ comments, made adjustments in areas of discrepancy, and produced a revised version that reflected the comments of all three translators. The reviewers were advised to produce semantic, idiomatic, experiential, and conceptual equivalence between the new Brazilian (JSE-Br) version and the original (JSE) and Portuguese (JSE-Pt) versions [14,16]. We sent this revised version to a native English translator, who back-translated the document. Both the original and Portuguese authors received the back-translated version so that final changes could be implemented.

#### Phase 2: Pretesting

After having been proofread, the JSE-Br (see Additional File: 1) was tested on 39 fifth-year medical students (86.7% of the total) during their internal medicine clerkship OSCE in September 2010. These 39 students also underwent a cognitive debriefing to confirm their comprehension of the instrument items.

#### Phase 3: Validation

Among the total of 319 fifth- and sixth-year medical students during the final-year OSCE in October 2011, 299 students participated in the study (93.7%). Three students (1%) were excluded because they did not respond to more than four items of the JSE [6]. Only 149 (49.83% of respondents) reported their gender: 92 male, 61.75%; 57 female, 38.25%).

### Statistical analysis

Internal consistency reliability was determined using Cronbach’s alpha coefficient. Values greater than 0.7 were considered satisfactory [19].

We assessed the construct validity by means of exploratory factor analysis: Bartlett’s test of sphericity and the Kaiser-Meyer-Olkin measure of sampling adequacy were used to check for the appropriateness of the factor analysis. We ran a principal component analysis (PCA) with varimax rotation. Eigenvalues greater than 1.5 were required to retain component factors, and factor loadings of 0.35 or greater were considered satisfactory for the interpretation of the factor structure with a sample size of 250–350 subjects [19]. Communalities (proportion of variance of the variable that is accounted for by the common factors) were calculated to assess the factor structure. We performed descriptive analyses on all items and determined the item-total correlation (the degree to which each item correlates to the total score) using Pearson’s coefficient. Gender groups were compared (*t* test) to check for known-group validity.

## Results

**Figure 1:**
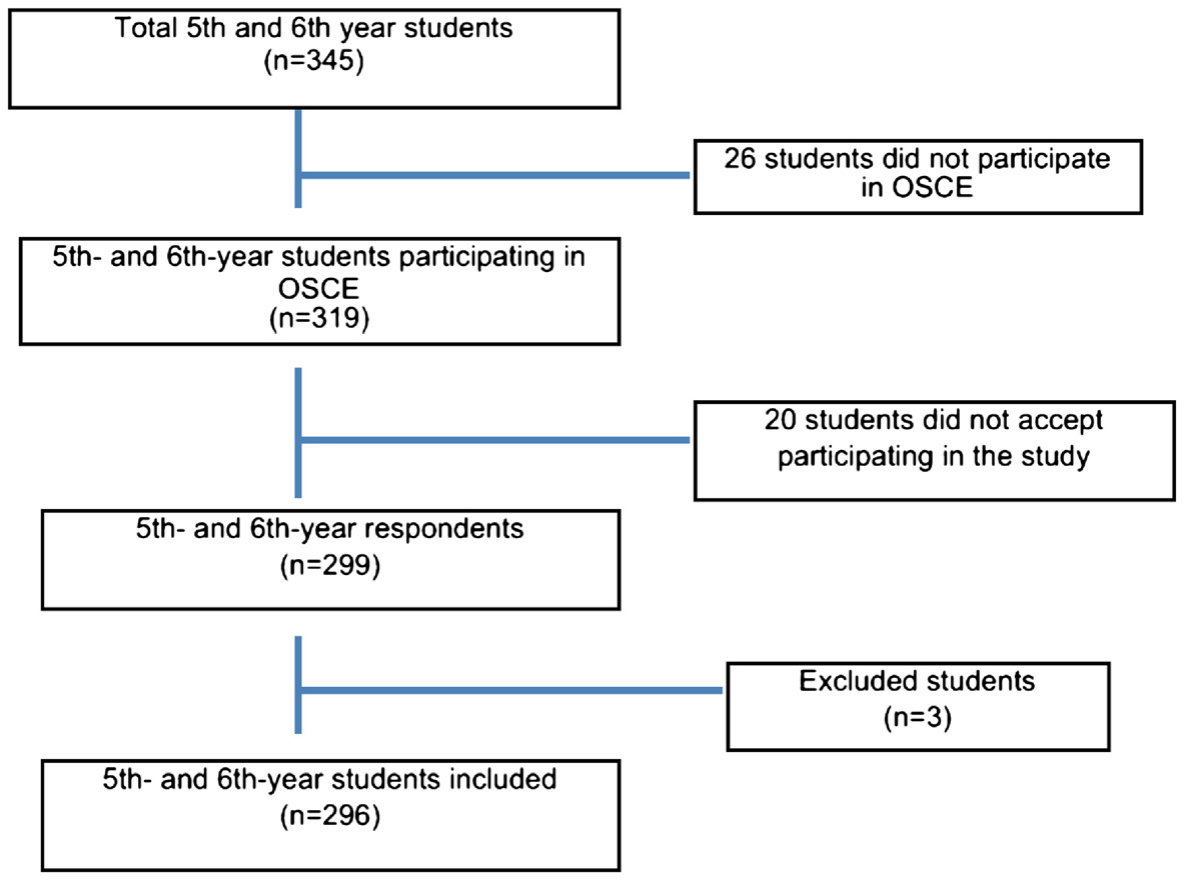
Response rate among fifth- and sixth-year medical students, October, 2011.

As noted above, the student response rate during the validation phase was 93.7% (Figure 1). Cronbach’s alpha coefficient for the scale was 0.84. Alpha values for each factor are shown in Table 1.

**Table 1:** **Factor pattern coefficients, mean and SD, communalities (h**^**2**^**) for principal component analysis with Varimax rotation and corrected item-total correlations on the 20 items of the JSE-Br (n = 296)**^**†**^

**Item**	**F1**	**F2**	**F3**	**Mean (SD)**	***h***^***2***^	***r***_***i-t***_
11. Patients’ illnesses can be cured only by medical or surgical treatment; therefore, physicians’ emotional ties with their patients do not have a significant influence in medical or surgical treatment	**0.77**	0.08	0.11	6.31 (1.04)	0.61	0.60
14. I believe that emotion has no place in the treatment of medical illness	**0.76**	0.16	0.08	6.38 (1.22)	0.61	0.59
12. Asking patients about what is happening in their personal lives is not helpful in understanding their physical complaints	**0.69**	0.18	0.01	6.09 (1.17)	0.51	0.49
16. Physicians’ understanding of the emotional status of their patients as well as that of their families is an important component of the physician–patient relationship	**0.68**	0.00	0.25	6.17 (0.97)	0.52	0.58
7. Attention to patients’ emotions is not important in history taking	**0.65**	0.03	0.20	6.34 (1.06)	0.46	0.55
8. Attentiveness to patients’ personal experiences does not influence treatment outcomes	**0.60**	−0.01	0.37	6.03 (1.21)	0.50	0.61
20. I believe that empathy is an important therapeutic factor in medical treatment	**0.60**	0.06	0.33	6.32 (0.85)	0.47	0.58
19. I do not enjoy reading nonmedical literature or that of the arts	**0.51**	−0.05	−0.09	6.38 (1.20)	0.27	0.27
15. Empathy is a therapeutic skill without which the physician’s success is limited	**0.44**	−0.04	0.40	5.95 (1.18)	0.35	0.48
2. Patients feel better when their physicians understand their feelings	**0.35**	−0.25	0.32	6.55 (0.93)	0.28	0.35
1. Physicians’ understanding of their patients’ feelings and the feelings of their patients’ families does not influence medical or surgical treatment	**0.30**	0.05	0.12	6.24 (1.42)	0.11	0.27
3. It is difficult for a physician to view things from the patient’s perspective	0.08	**0.85**	0.07	3.97 (1.44)	0.74	0.23
6. Because people are different, it is difficult to see things from the patient’s perspective	0.09	**0.85**	0.05	4.07 (1.53)	0.73	0.21
5. A physician’s sense of humor contributes to a better clinical outcome	0.02	0.04	**0.69**	5.48 (1.28)	0.48	0.35
9. Physicians should try to stand in their patients’ shoes when providing care to them	0.22	0.18	**0.63**	5.71 (1.36)	0.48	0.50
13. Physicians should try to understand what is going on in their patients’ minds by paying attention to their nonverbal cues and body language	0.47	0.09	**0.58**	5.74 (1.22)	0.56	0.66
10. Patients value a physician’s understanding of their feelings, which is therapeutic in its own right	0.33	−0.06	**0.53**	5.82 (1.14)	0.40	0.47
4. Understanding body language is as important as verbal communication in physician-patient relationships	0.36	−0.06	**0.52**	6.06 (1.04)	0.40	0.50
17. Physicians should try to think like their patients to render better care	0.02	0.16	**0.67**	5.18 (1.45)	0.47	0.38
18. Physicians should not allow themselves to be influenced by strong personal bonds between their patients and their family members	0.22	−0.05	**0.34**	4.17 (1.51)	0.16	0.30
Eigenvalue	5.99	1.60	1.55			
% of variance	29.98	8.04	7.76			
Cronbach’s alpha	0.83	0.73	0.74			

^†^Factor labels are as follows: F1, Factor 1 (Compassionate Care); F2, Factor 2 (Standing in the Patient’s Shoes); F3, Factor 3 (Perspective Taking).

### Principal component analysis

The Kaiser-Meyer-Olkin analysis yielded an index of 0.88, and Bartlett’s test of sphericity gave *X*^*2*^ = 1772.00 (*p* = 0.00); these indicate the appropriateness of the data for PCA. Three factors with eigenvalues ≥1.5 were extracted by PCA and accounted for 45.48% of the overall variance. As shown in Table 1, the first factor (denoted as Compassionate Care) accounted for 29.98% of the total variance, and it included 11 items with factor loadings ≥0.30. The second factor (Standing in the Patient’s Shoes) accounted for 8.04% of the variance, and it consisted of two negative items with factor loadings ≥0.85. The third factor (Perspective Taking) accounted for 7.76% of the variance, and comprised seven items with factor loadings ≥0.30.

Items 1 and 18 had factor loadings ≤0.35. Items 2 and 15 had similar factor loadings for factors 1 and 2. Communality values were lower than 0.40 for items 1, 2, 15, 18, and 19.

### Psychometric properties of the JSE

#### Item descriptive statistics and correlations

The mean total score for the JSE was 114.95 (SD = 12.41). Mean item scores ranged from 3.97 (SD = 1.44) to 6.55 (SD = 0.93) (Table 1). The lowest score was observed for item 3 (“It is difficult for a physician to view things from the patient’s perspective”). Students scored highest on item 2 (“Patients feel better when their physicians understand their feelings”).

All items were positively correlated with their corresponding factors and subscales. The correlation coefficients were all positive and ranged from 0.50 to 0.89 (*p* ≤ 0.001; Table 1).

The subscales Compassionate Care and Perspective Taking were strongly correlated (*r* = 0.61; *p* ≤ 0.001). The subscale Compassionate Care was also correlated to Standing in the Patient’s Shoes (*r* = 0.16; *p* ≤ 0.05; Table 2).

**Table 2:** **Pearson’s correlation coefficients among the Jefferson Scale of Empathy (JSE) subscales (*n*** **= 296)**

**Subscale**	**Compassionate care**	**Standing in the patient’s shoes**	**Perspective taking**	**Total score**
Compassionate Care		0.16^†^	0.59^†^	0.90^†^
Standing in the Patient’s Shoes	0.16^†^		0.15^†^	0.38^†^
Perspective Taking	0.59^††^	0.15^†^		0.84^†^

^†^p ≤ 0.05; ^†^^†^p ≤ 0.01.

### Known-group validity

Although female students had higher mean empathy scores than males (116.47 versus 113.79), this difference was not statistically significant (*p* = 0.21; Table 3).

**Table 3:** **Gender comparisons among Jefferson Scale of Empathy (JSE) subscales (*n*** **= 149)**^**†**^

	**Male (*n*** **= 92)**	**Female (*n*** **= 57)**	***t***	***p***^**††**^
	**Mean (SD)**	**Mean (SD)**		
Total score	113.84 (12.68)	116.54 (12.81)	−1.26	0.21
Compassionate Care	67.89 (7.67)	69.80 (7.62)	−1.49	0.13
Standing in the Patient’s Shoes	7.92 (2.62)	7.78 (2.69)	0.31	0.75
Perspective Taking	38.03 (5.43)	38.95 (6.30)	−0.95	0.35

^†^147 students did not report their gender; ^††^*t* test.

## Discussion

This study aimed to assess the reliability and validity of the Brazilian version of the JSE. Its reliability was supported by a high internal consistency value. Our results confirm the international robustness of the scale: Cronbach’s coefficients were consistently high [8-12,20,21].

These results also support the construct validity of the JSE-Br because PCA was able to replicate the three factors that emerged in the original sample [3]. Previous studies have also confirmed the construct validity of the JSE as a three-domain measure of empathy (Perspective Taking, Compassionate Care, and Standing in the Patient’s Shoes) in Mexico [8], Korea [21], Iran [11], and the United Kingdom [12].

Interestingly, the first factor that emerged in the PCA of our study was the Compassionate Care component. This result is in accordance with Iranian findings [11], but it is in contrast with the original scale factor structure, in which the Perspective Taking component was the major dimension [3]. This difference could be attributed not only to cultural issues but also to religious issues. In Brazil, Christianity is the main religion, and such characteristics as compassion and charity are considered fundamental in this country. Another explanation for this result may be related to differences between the United States and Brazil concerning bioethical issues in doctor-patient relationships. In the United States, discussions on power and the patient’s autonomy have taken place since the 1960s [22]. In Brazil, the Hippocratic paternalism in doctor-patient relationships has been questioned only after fairly recent reforms to the Medical Ethics Code in the late 1990s [23], and paternalistic behavior is still common among Brazilian doctors and students. Such considerations must be taken into account when interpreting the importance of compassion in Brazilian culture.

Another possible interpretation of our results relates to the definition of compassion. If compassion is understood to be “an overlap between cognition and emotion” [3], it may be inferred that in our sample, emotions played the most important role in utilizing humanistic skills in the patient-care setting. Brazilian students seem to consider empathy a largely emotional construct.

Similarly, the Standing in the Patients’ Shoes component, the second factor in our results, may also reflect the importance of compassion among Brazilian medical students. This component appeared third in the original scale.

However, the Perspective Taking component was the third factor in our analyses. This domain is related to cognitive skills: information processing, reasoning, appraisal, and communicating empathy [3]. Cultural differences may partially explain these results, but curricular issues also have to be taken into account. Brazilian students are rarely exposed to such skills in their medical training in most of Brazil’s medical schools. This area of competence is usually practiced at isolated moments of the medical curriculum related to the interdisciplinary field of humanities, patient safety [24], and bioethics. This presentation format in the curriculum may erroneously suggest to students that empathy is an exclusively inner characteristic or a natural disposition [25] rather than a multidimensional competence to be taught, trained, and assessed [24,26].

Our results showed that item 1 (“Physicians’ understanding of their patients’ feelings and the feelings of their patients’ families does not influence medical or surgical treatment”) yielded low factor loading (*r* = 0.30) and communality (*h*^*2*^ = 0.11) values. This pattern was similar to that in the UK study, which was conducted among 853 medical students (*r* < 0.40; *h*^*2*^ = 0.15) [12]. Although low communality values may indicate the need for larger samples, it is unlikely that our result is due to sample size. The number of respondents in our study exceeded the adequate proportion of 10 subjects per observed variable [19]. Interestingly, in the Portuguese validation, item 1 was excluded owing to null variation in the confirmatory factor analysis [13]. Further studies with the JSE-Br are needed to explain these results.

In our sample, item 18 (“Physicians should not allow themselves to be influenced by strong personal bonds between their patients and their family members”) also presented low values of factor loading and communality (*r* = 0.34; *h*^*2*^ = 0.16) in the Perspective Taking component. Mexican [8] and Japanese [10] validation studies reported similar results for item 18 factor loadings (*r* = 0.25 and *r* = 0.36, respectively). In contrast to our study, this item was originally factored in the Compassionate Care component in Mexico and Japan [20]. In the Mexican [8] and Japanese [10] samples, item 18 was factored in the Ability to Stand in the Patient’s Shoes component. Scores from these low-communality items must be assessed with caution in Brazilian samples until further studies are conducted.

Our results showed that items 2 (“Patients feel better when their physicians understand their feelings”) and 15 (“Empathy is a therapeutic skill without which the physician’s success is limited”) may be considered factorially complex because they showed similar loadings in both the Compassionate Care and Perspective Taking components. Indeed, these components are strongly associated, as suggested by the significant and large correlation coefficient between these two subscales. In the original validation studies [20], these items were factored in the Perspective Taking subscale. In our context, these items were categorized under Compassionate Care. These results reinforce our hypothesis that Brazilian students seem to understand empathy as an emotional rather than a cognitive construct.

Similar to the results with Italian [9], Korean [21], and Iranian [11] medical students, our data failed to demonstrate higher empathy scores among female students, as measured by the JSE. This result could be due to sampling bias because only 49% of the respondents reported their gender in our study. However, gender differences have been reported in several studies among medical trainees, with the results favoring female students in terms of higher empathy [8,10,12]. Such controversies remain to be elucidated: current neuroscience studies investigate not only intrinsic factors related to empathy, such as genetics [27] and brain networks [28,29], but also extrinsic factors, such as social values and culture [30], that could possibly explain gender differences. Are these differences a matter of gender? This question remains unanswered.

There were certain methodological limitations to our study. Although it may have been valuable to our study, we did not test the concurrent validity of the JSE with other measures of empathy. We attempted to mitigate the students’ response burden during OSCE evaluations and chose to test validity through factor analysis and known-group validity. Medical students were recruited by convenience, so sampling bias must be taken into account. However, our high response rate may have moderated this limitation, and our sample is probably representative of the study population. We were unable to explain items with low factor coefficients and communalities. Further studies with confirmatory factor analyses in different datasets are needed to better understand item behavior within the JSE. Additionally, the low rate of respondents reporting gender may have limited our conclusions regarding gender differences.

## Conclusions

Our study followed rigorous techniques of adaptation to produce a robust, psychometrically sound measure of empathy that was closely matched to the original instrument. The use of a validated measure may help evaluate strategies for learning empathy and consolidate its association with clinical performance in future studies. Moreover, the use of the JSE-Br in national and cross-cultural studies is promising: understanding intercultural differences may promote more effective educational interventions and transcend boundaries in medical education.

## Competing interests

The authors declared that they have no competing interests.

## Authors’ contributions

HBMSP conceived the study, participated in data collection, performed statistical analysis, and drafted and revised the manuscript. RMDG conceived the study, participated in data collection, performed statistical analysis, and revised the manuscript. ICT conceived the study, participated in data collection, and revised the manuscript. RMCP performed statistical analysis, and drafted and revised the manuscript. MAM conceived the study and revised the manuscript. All authors read and approved the final manuscript.

## Pre-publication history

The pre-publication history for this paper can be accessed here:

http://www.biomedcentral.com/1472-6920/12/73/prepub

## Supplementary Material

Additional file 1 **Brazilian version of the Jefferson Scale of Empathy (JSE-Br).** Portuguese(Brazil)_S_(HParo).pdf - Escala Jefferson de Empatia Médica – Versão para Estudantes. (PDF 96 kb) Click here for file
