# Supplementary material for: Brazilian version of the Jefferson Scale of Empathy: psychometric properties and factor analysis
Source: BMC Med Educ. 2012 Aug 9;12:73. doi: 10.1186/1472-6920-12-73 (PMC3528616; doi:10.1186/1472-6920-12-73)
Supplement: Additional file 1 — Brazilian version of the Jefferson Scale of Empathy (JSE-Br). Portuguese(Brazil)_S_(HParo).pdf - Escala Jefferson de Empatia Médica – Versão para Estudantes. (PDF 96 kb) [file 1472-6920-12-73-S1.pdf]

Escala Jefferson de Empatia Médica – Versão para Estudantes

Por favor, indique o seu nível de concordância com as seguintes afirmações:

(Assinale a opção escolhida na seguinte escala com um [x] ; em caso de erro, preencha por completo o quadrado ■ e assinale com um [x] a opção correta)

Discordo fortemente					Concordo fortemente	
1	2	3	4	5	6	7

	1	2	3	4	5	6	7
1. A compreensão dos médicos em relação aos sentimentos dos seus pacientes e de seus familiares não tem influência no tratamento clínico ou cirúrgico.	□	□	□	□	□	□	□
2. Os pacientes sentem-se melhor quando os médicos compreendem os seus sentimentos.	□	□	□	□	□	□	□
3. É difícil para um médico ver as coisas na perspectiva dos pacientes.	□	□	□	□	□	□	□
4. Compreender a linguagem não verbal (corporal) é tão importante quanto compreender a linguagem verbal nas relações médico-paciente.	□	□	□	□	□	□	□
5. O senso de humor de um médico contribui para resultados clínicos melhores.	□	□	□	□	□	□	□
6. Considerando que as pessoas são diferentes, é difícil ver as coisas na perspectiva dos pacientes.	□	□	□	□	□	□	□
7. Prestar atenção às emoções dos pacientes não é importante ao se obter a história clínica.	□	□	□	□	□	□	□
8. A atenção às experiências pessoais dos pacientes não influencia o resultado dos tratamentos.	□	□	□	□	□	□	□
9. Os médicos deveriam tentar colocar-se no lugar dos seus pacientes quando estão cuidando deles.	□	□	□	□	□	□	□
10. Os pacientes valorizam a compreensão que o médico tem dos seus sentimentos, o que é terapêutico por si próprio.	□	□	□	□	□	□	□
11. As doenças dos pacientes só podem ser curadas por meio de tratamentos médicos ou cirúrgicos; assim, os laços emocionais estabelecidos entre médicos e seus pacientes não têm influência significativa no tratamento clínico ou cirúrgico.	□	□	□	□	□	□	□
12. Fazer perguntas aos pacientes sobre o que se passa na sua vida privada não ajuda na compreensão das suas queixas físicas.	□	□	□	□	□	□	□
13. Os médicos deviam tentar compreender o que se passa na cabeça dos seus pacientes, prestando mais atenção aos sinais não verbais e à sua linguagem corporal.	□	□	□	□	□	□	□
14. Eu acredito que as emoções não têm qualquer participação no tratamento das doenças.	□	□	□	□	□	□	□
15. A empatia é uma habilidade terapêutica sem a qual o sucesso do médico é limitado.	□	□	□	□	□	□	□
16. A compreensão dos médicos acerca do estado emocional dos seus pacientes e das famílias dos seus pacientes é um componente importante da relação médico-paciente.	□	□	□	□	□	□	□
17. Os médicos deveriam tentar pensar como os seus pacientes para prestarem melhores cuidados.	□	□	□	□	□	□	□
18. Os médicos não deveriam se deixar influenciar pela existência de fortes relações pessoais com os seus pacientes e as famílias.	□	□	□	□	□	□	□
19. Não aprecio literatura não médica ou outras formas de arte.	□	□	□	□	□	□	□
20. Eu acredito que a empatia é um fator terapêutico importante no tratamento médico.	□	□	□	□	□	□	□

Portuguese (Brazil) translation by Helena Paro, Iolanda Tibério and Renata Daud-Gallotti, University of São Paulo, Brazil

For permission to use the scale contact: Empathy.Scales@Jefferson.edu

© Jefferson Medical College, All rights reserved
